# The Ubiquitin-Proteasome System Does Not Regulate the Degradation of Porcine β-Microseminoprotein during Sperm Capacitation

**DOI:** 10.3390/ijms21114151

**Published:** 2020-06-10

**Authors:** Lucie Tumova, Michal Zigo, Peter Sutovsky, Marketa Sedmikova, Pavla Postlerova

**Affiliations:** 1 Department of Veterinary Sciences, Faculty of Agrobiology, Food and Natural Resources, Czech University of Life Sciences Prague, 165 00 Prague, Czech Republic; tumovalucie@af.czu.cz (L.T.); sedmikova@af.czu.cz (M.S.); 2 Division of Animal Sciences, University of Missouri, Columbia, MO 65211, USA; zigom@missouri.edu (M.Z.); SutovskyP@missouri.edu (P.S.); 3 Department of Obstetrics, Gynecology & Women’s Health, University of Missouri, Columbia, MO 65211, USA; 4 Laboratory of Reproductive Biology, Institute of Biotechnology of the Czech Academy of Sciences, BIOCEV, 252 50 Vestec, Czech Republic

**Keywords:** boar, spermatozoa, capacitation, β-microseminoprotein, MSMB, PSP94, ubiquitin-proteasome system

## Abstract

Sperm capacitation, one of the key events during successful fertilization, is associated with extensive structural and functional sperm remodeling, beginning with the modification of protein composition within the sperm plasma membrane. The ubiquitin-proteasome system (UPS), a multiprotein complex responsible for protein degradation and turnover, participates in capacitation events. Previous studies showed that capacitation-induced shedding of the seminal plasma proteins such as SPINK2, AQN1, and DQH from the sperm surface is regulated by UPS. Alterations in the sperm surface protein composition also relate to the porcine β-microseminoprotein (MSMB/PSP94), seminal plasma protein known as immunoglobulin-binding factor, and motility inhibitor. MSMB was detected in the acrosomal region as well as the flagellum of ejaculated boar spermatozoa, while the signal disappeared from the acrosomal region after in vitro capacitation (IVC). The involvement of UPS in the MSMB degradation during sperm IVC was studied using proteasomal interference and ubiquitin-activating enzyme (E1) inhibiting conditions by image-based flow cytometry and Western blot detection. Our results showed no accumulation of porcine MSMB either under proteasomal inhibition or under E1 inhibiting conditions. In addition, the immunoprecipitation study did not detect any ubiquitination of sperm MSMB nor was MSMB detected in the affinity-purified fraction containing ubiquitinated sperm proteins. Based on our results, we conclude that UPS does not appear to be the regulatory mechanism in the case of MSMB and opening new questions for further studies. Thus, the capacitation-induced processing of seminal plasma proteins on the sperm surface may be more complex than previously thought, employing multiple proteolytic systems in a non-redundant manner.

## 1. Introduction

To acquire fertilizing ability, mammalian spermatozoa undergo extensive post-testicular maturation and sub-cellular, molecular changes. One of the most important events is sperm capacitation in the female reproductive tract [1]. Capacitation is a complex process that endows spermatozoa with the potential to bind zona pellucida and the ability to undergo acrosomal exocytosis, further to penetrate zona pellucida, and to fuse with an oocyte [2]. 

Seminal plasma proteins are involved in the process of capacitation, functioning as decapacitation factors that maintain ejaculated spermatozoa viability within the female reproductive system. These proteins bind to the sperm surface during ejaculation and are involved in the formation of the oviductal sperm reservoir and zona pellucida binding [3,4]. During capacitation, many changes occur in the sperm plasma membrane and the removal of decapacitating factors leading to the rearrangement of sperm surface proteins [2,5]. One of these proteins undergoing such changes during capacitation is a β-microseminoprotein (MSMB), also known as prostatic secretory protein (PSP94), immunoglobulin-binding factor [6], sperm motility inhibitor [7], or prostatic inhibin peptide [8]. MSMB was originally identified in human seminal plasma [9] and has also been reported in several other species [8,10,11]. Human MSMB is present in a high concentration in prostatic secretions [11]; however, it has also been found in other bodily fluids. The precise role of MSMB is still to be elucidated. It was suggested that in humans it may serve as an immunoglobulin-binding factor [6] and as a marker of gastric cancer diseases [12]. Additionally, it has been ascertained to suppress prostatic tumor cell growth [8] and to protect prostatic cells from pathogens [13]. Primarily, human MSMB plays a very important role as a marker of prostate cancer [14,15,16]. Sperm MSMB is probably involved in the interactions between spermatozoa and zona pellucida at fertilization as well as in the regulation of sperm hyperactivation at the time of sperm capacitation [11]. In a more recent study, sperm MSMB has been found to associate with CRISPs (cysteine-rich secretory proteins) [17] implicated in gamete binding and fusion [18].

Porcine β-microseminoprotein shows about 50% homology to human MSMB [10]. In previous studies, porcine MSMB has been found mainly in secretions and epithelia of the prostate gland [19,20,21], as well as in germ cells inside the testicular seminiferous tubules, epididymal fluid and epithelium, Cowper’s glands, urethral gland, and seminal vesicles. In addition, MSMB has also been detected in brain, kidney, and muscle tissues [22]. Similar to humans, porcine MSMB is synonymous with immunoglobulin-binding factor in seminal fluid and may affect local immunity [6]. Porcine MSMB also acts as a sperm motility inhibitor through the inhibition of sodium-potassium pumps [7,10,23]. As mentioned above, porcine MSMB has been detected in many reproductive tissues, thus suggesting multiple roles in the reproductive process. The localization of MSMB in the head and flagellum of porcine spermatozoa was reported previously as well as its post-capacitation fate [22].

The mechanism by which MSMB is lost from the sperm surface during capacitation is still unknown. The ubiquitin-proteasome system (UPS) was implicated in the regulation of other seminal plasma proteins of the sperm surface, such as SPINK2, AQN1 [24], and DQH [25]. UPS is an important regulatory mechanism in most cells that provides substrate-specific proteolysis of about 75% of all eukaryotic proteins [26] including the regulation of the fertilization process [27,28,29]. UPS plays an important role as a control mechanism of sperm quality [30]. In ejaculated spermatozoa, UPS first regulates capacitation [29], and subsequently sperm-zona pellucida penetration [31]. UPS is complex, multi-enzyme machinery that is composed of three main ubiquitinating enzymes—E1 activating enzyme (UBA1), E2 conjugating enzyme (UBC), and E3 ubiquitin ligase (UBE), and 26S proteasome as the endpoint protease [28]. It is significantly involved in the recycling of cellular proteins and regulation of signaling pathways through the post-translational modification of proteins, called protein ubiquitination. Ubiquitin specifically labels proteins designated for degradation by 26S proteasome, while it may also channel protein aggregates and organelles towards the autophagic pathway. The canonical 26S proteasome is composed of a 20S proteolytic core and a 19S regulatory particle, capping the 20S barrel at one or both ends. The 19S particle is responsible for recognition of the polyubiquitin chain, protein unfolding, deubiquitination, and presentation of the unfolded protein to the 20S core for proteolytic degradation [26,28].

Previous studies were dedicated to elucidating the role of UPS in sperm capacitation. Several boar sperm surface proteins were found to copurify with sperm proteasomes, making them likely targets of sperms’ resident UPS [32]. Another study has shown that UPS plays a crucial part in the removal of sperm surface proteins and the plasma membrane remodeling during human sperm capacitation [33]. Degradation of A-kinase-anchoring protein by UPS proved to be crucial for successful hyperactivation [34]. Our previous results have demonstrated that during capacitation, the UPS participates in compartmentalization and processing of proteins such as lactadherin MFGE8, ADAM5, and ACRBP, and numerous other candidates [35]. UPS has also been implicated in the de-aggregation of spermadhesins and processing of DQH protein on the sperm surface [25], and in the recently discovered, capacitation-induced zinc ion efflux from spermatozoa [36]. Building on our previous results that some proteins are removed from the sperm surface during IVC via their ubiquitination [24,25,35], we hypothesized that yet another seminal plasma protein, MSMB, may be a target of UPS during sperm capacitation. This study was therefore designed to examine this possibility to further explore the complex role of UPS in sperm capacitation.

## 2. Results

The degradation of porcine β-microseminoprotein (MSMB) under proteasomal inhibition and E1 inhibiting conditions during in vitro sperm capacitation was studied by flow cytometric analysis and Western blot detection. Additionally, we performed indirect immunofluorescence staining to monitor the MSMB localization in boar spermatozoa and changes in the anti-MSMB antibody labeling on sperm after in vitro capacitation (IVC).

### 2.1. Localization and Changes of MSMB in Boar Spermatozoa during In Vitro Capacitation

**Figure 1:**
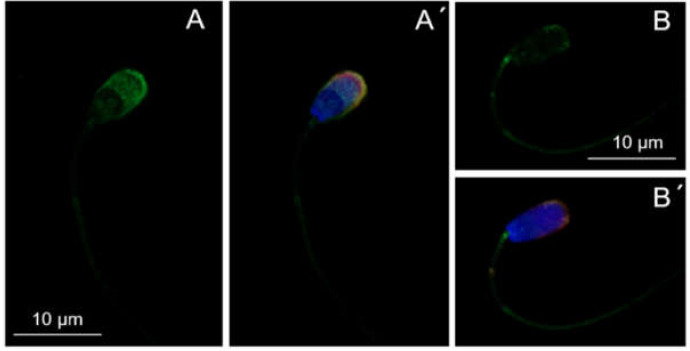
Localization of porcine MSMB in ejaculated (**A**,**A**’) and in vitro capacitated (**B**,**B´**) spermatozoa with a specific polyclonal anti-MSMB antibody (green) by indirect immunofluorescent microscopy. Nucleus was counterstained with DAPI (4′,6-diamidino-2-phenylindole) (blue) and acrosome with PNA (Peanut agglutinin) lectin (red).

MSMB was localized in ejaculated as well as in IVC spermatozoa. Ejaculated spermatozoa showed a high labeling intensity of MSMB in the acrosomal region (Figure 1A), whereas the signal intensity was reduced in IVC spermatozoa (Figure 1B). Fluorescent intensity was not significantly different between individual IVC spermatozoa treatment groups, i.e., non-inhibited, proteasomally-inhibited, E1-inhibited, and vehicle control (Figure S1).

**Figure 2:**
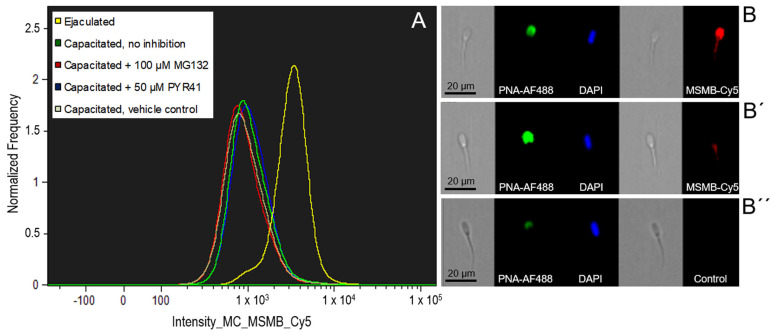
A representative flow cytometric histogram of MSMB changes during sperm in vitro capacitation without or under proteasomal (100 µM MG132)/E1 (50 µM PYR41) inhibiting conditions including vehicle control. The mean value of all flow cytometric measurements showed a higher fluorescence intensity in ejaculated spermatozoa (**A**). Representative image galleries of ejaculated spermatozoa (**B**), capacitated spermatozoa (**B’**), and negative control spermatozoa incubated with non-immune serum in place of anti-MSMB antibody (**B”**). Nuclei were counterstained with DAPI (blue); acrosomal integrity was monitored with lectin PNA (green) and binding of MSMB-Cy5 antibody (red). Every flow cytometric run represents 10,000 events. The experiment was replicated four times.

**Figure 3:**
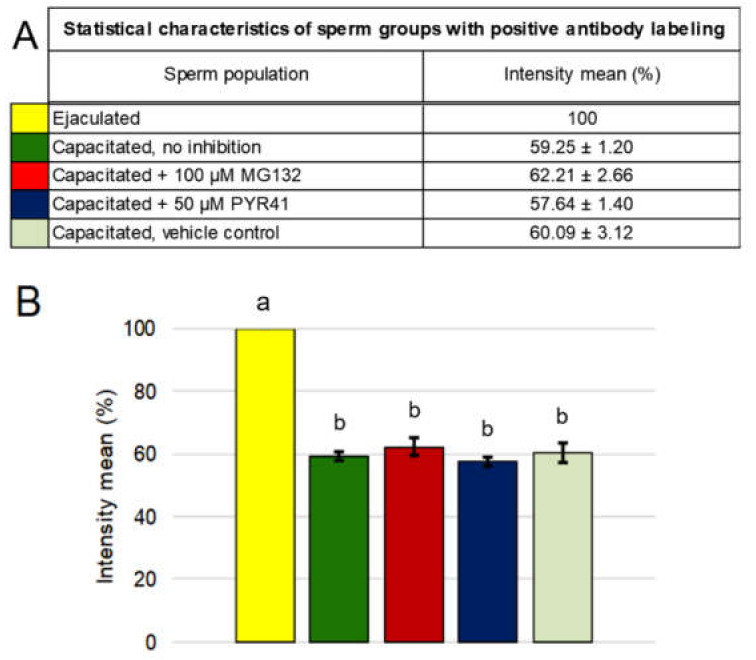
Quantification of the MSMB removal during in vitro capacitation (IVC). The baseline fluorescent intensity mean of ejaculated spermatozoa was defined as 100%, to which the other IVC sperm groups were compared. (**A**) The decrease in fluorescent intensity mean in IVC spermatozoa treatment groups, i.e., non-inhibited, proteasomally-inhibited, E1-inhibited, and vehicle control. (**B**) Graphic representation of fluorescent intensity means in all treatment groups. Results are presented as the mean ± SD of four independent biological replicates. Statistical significance (*p* < 0.05) is indicated by superscripts.

Image-based flow cytometry (IBFC) was performed to monitor the changes of MSMB during IVC. Spermatozoa with intact acrosomes were gated and subjected to further analysis. The fluorescence intensity histogram of MSMB labeling showed the presence of two sperm populations (Figure 2A). The majority of ejaculated spermatozoa was present in the population with high fluorescence intensity, and intensive labeling in the acrosomal area (Figure 2B). Such labeling was significantly reduced in spermatozoa after IVC (Figure 2B’), resulting in the second population and a corresponding IBFC histogram peak with lower median fluorescence intensity (Figure 2A). The loss of fluorescence intensity signifies the removal of MSMB from the acrosomal region during IVC. The mean value of the antibody-induced fluorescent staining referred to as “intensity mean” was designated in ejaculated spermatozoa as 100%, with other IVC sperm treatment groups compared to it based on their intensity means (Figure 3A). MSMB fluorescence intensity mean significantly decreased (*p* < 0.05) after IVC in non-inhibited spermatozoa, to 59.25 ± 1.20% when compared to ejaculated spermatozoa (Figure 3). While IVC spermatozoa under proteasomal inhibition (100 µM MG132) showed the fluorescence intensity mean of MSMB at 62.21 ± 2.66%, capacitated spermatozoa under ubiquitin-activating enzyme (E1) inhibition by 50 µM PYR41 demonstrated the fluorescence intensity mean of MSMB equal to 57.64 ± 1.40%. No statistical difference (*p* > 0.05) was found between the vehicle control group 60.09 ± 3.12 % and other IVC capacitated treatment groups (Figure 3B). 

### 2.2. Detection of MSMB in Boar Sperm Extracts

**Figure 4:**
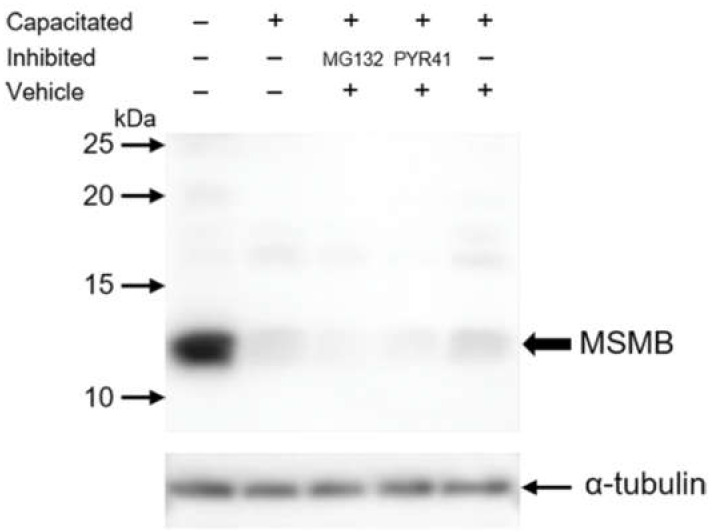
Western blot detection of porcine MSMB with specific polyclonal anti-MSMB antibody in the protein extracts from ejaculated and IVC spermatozoa under non-inhibiting, proteasomally-inhibited (100 µM MG132), and E1-inhibited conditions (50 µM PYR41), also including vehicle control (DMSO). The black arrow indicates the expected immunoreactive band of MSMB of approximately 12 kDa. Equal protein loads were confirmed by monoclonal antibody anti-α-tubulin DM1A. SDS-PAGE was run under reducing conditions and the experiment was replicated four times, see Figure 5 for densitometric quantification.

Western blot detection under reducing conditions was used to detect and quantify a 12 kDa MSMB immunoreactive band in boar sperm protein extract in all sperm treatment groups (Figure 4). In protein extract of ejaculated spermatozoa, the amount of MSMB was higher than in spermatozoa capacitated in in vitro conditions. To verify the protein load of each sample and to normalize MSMB content, membranes were reprobed with an anti-α-tubulin antibody.

**Figure 5:**
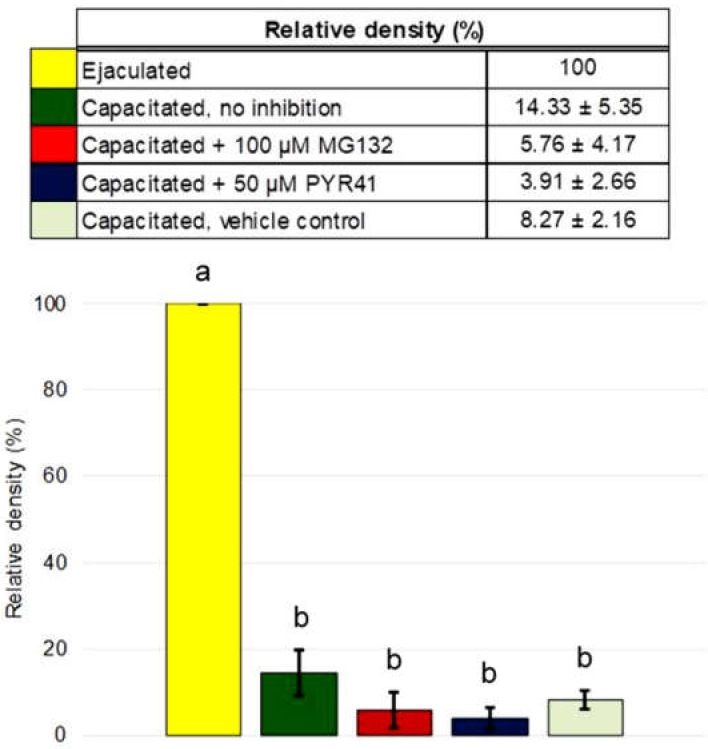
Densitometric quantification of 12 kDa immunoreactive MSMB bands from Figure 4 in protein extracts of ejaculated spermatozoa and all capacitated sperm treatment groups. The relative density of MSMB in the blot was calculated as the ratio of the optical density of anti- MSMB and anti-α-tubulin antibodies; the MSMB amount in the ejaculated sperm sample was defined as 100%, and all IVC sperm treatment groups were compared to ejaculated spermatozoa. Results are presented as the mean ± SD of four independent biological replicates. Statistical significance (*p* < 0.05) is indicated by superscripts.

The MSMB content in the ejaculated sperm sample was defined as 100% and all IVC sperm treatment groups were compared relative to ejaculated spermatozoa (Figure 5). In non-inhibited IVC spermatozoa, the amount of MSMB was significantly decreased (14.33 ± 5.35%) when compared to ejaculated spermatozoa. In IVC spermatozoa under 100 μM MG132 proteasomal inhibition, the amount of MSMB was decreased to 5.76 ± 4.17%, while under ubiquitin-activating enzyme (E1) inhibition with 50 μM PYR41, the amount of MSMB declined to 3.91 ± 2.66%. In vehicle control, the amount of MSMB decreased to 8.27 ± 2.16% after IVC (Figure 4 and Figure 5). A statistically significant difference was only found in the relative density between ejaculated and in vitro capacitated sperm groups, regardless of the treatment (*p* < 0.05). No statistical significance of MSMB accumulation was found within different treatment groups of IVC spermatozoa (*p* > 0.05, Figure 5).

### 2.3. Detection of Polyubiquitinated Forms of MSMB in Boar Sperm Extracts

**Figure 6:**
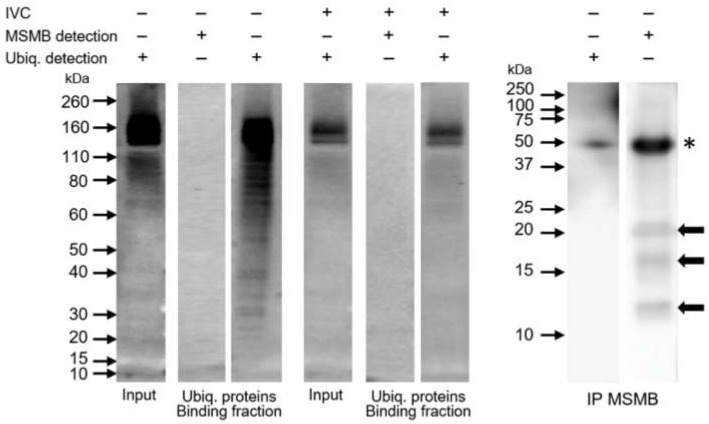
Immunodetection of porcine MSMB in (poly)ubiquitinated protein sample isolated from the extract of whole ejaculated and IVC spermatozoa using the Signal-Seeker^TM^ Ubiquitination Detection kit (Ubiq. proteins—Binding fraction) with control detection of polyubiquitinated proteins and reciprocal detection of ubiquitinated proteins in MSMB immunoprecipitate (IP MSMB) from the extract of ejaculated spermatozoa with control detection of MSMB. Black arrows show MSMB (12, 17 and 22 kDa); asterisk indicates antibody heavy chains.

Polyubiquitinated proteins from the extract of ejaculated and IVC spermatozoa were isolated using the Signal-Seeker^TM^ Ubiquitination Detection kit to monitor potential MSMB (poly)ubiquitination. The presence of polyubiquitinated proteins was confirmed by Western blotting with anti-ubiquitin antibody FK2; however, these isolates were void of MSMB in binding fraction containing ubiquitinated proteins (Figure 6, Ubiq. proteins—Binding fraction). The input sperm extracts before affinity purification were examined for ubiquitinated proteins using the FK2 antibody. Alternatively, MSMB was immunoprecipitated from the extract of ejaculated spermatozoa and the MSMB immunoprecipitate was probed for (poly)ubiquitinated proteins by FK2 antibody (Figure 6, IP MSMB). The presence of MSMB in the MSMB immunoprecipitate was confirmed by immunodetection of 12 kDa band, resp. 17 and 22 kDa (black arrows); however, no (poly)ubiquitinated proteins were detected with the FK2 antibody in the MSMB immunoprecipitate. Negative controls of immunoprecipitation was performed by incubation of ejaculated sperm extract with agarose-protein A/G beads only and using rabbit immunoglobulins instead of anti-MSMB antibody; neither polyubiquitinated proteins nor MSMB was detected in the eluate after incubation (Figure S2).

## 3. Discussion

Capacitation is a key event of the fertilization process, important for the final maturation of spermatozoa as they acquire fertilizing ability. Sperm capacitation encompasses many changes in the sperm plasma membrane, as well as the removal of decapacitating factors, leading to the rearrangement of sperm surface proteins necessary for sperm-ZP binding [2,5]. The ubiquitin-proteasome system (UPS), an instrument of substrate-specific protein degradation, may be involved in sperm surface protein removal during sperm capacitation, as some boar seminal plasma proteins have already been reported to copurify with sperm-borne proteasomes [32]. Furthermore, such proteins (SPINK2, AQN1, and DQH) accumulated after proteasomal inhibition during sperm in vitro capacitation (IVC) [25,35]. In this study, we aimed to explore the possibility that MSMB is ubiquitinated and degraded by UPS during sperm capacitation, as it was reported earlier that MSMB disappeared from the sperm surface after IVC [22].

Porcine MSMB is a relatively small protein that migrates electrophoretically under reducing condition at ~12 kDa [22]. In our previous study, we localized porcine MSMB in all male reproductive tissues with the highest abundance in the epithelium and prostate gland secretions. Additionally, porcine MSMB has been found on the surface of ejaculated spermatozoa, specifically in the acrosomal region of the sperm head, and the flagellum. During in vitro capacitation, a significant decrease of this protein has been observed, particularly in the acrosomal region. In addition, MSMB has been localized not only to the sperm surface but also inside the acrosome in IVC spermatozoa by using transmission electron microscopy, suggesting multiple roles in sperm maturation or fertilization. This finding may be the one piece of evidence that MSMB is involved in sperm-oocyte binding after acrosomal exocytosis [22], as previously proposed in humans [11].

Porcine MSMB is classified as a specific protein of seminal plasma and spermatozoa. As aforementioned, several pig sperm surface proteins have been copurified with sperm-borne proteasomes [32] and accumulated during IVC, which was linked to the IVC-induced change in the compartmentalization of these proteins [25,35]. In the present study, we observed the same fate for MSMB during IVC, yet the mechanism responsible for this protein’s loss, as well as the machinery responsible for it, is unknown. We, therefore, decided to explore the possibility of UPS engagement in MSMB removal as yet another target for UPS degradation during sperm capacitation. We capacitated spermatozoa under proteasome-inhibiting conditions to prevent degradation of potentially ubiquitinated MSMB, as well as under ubiquitin-activating enzyme (E1) inhibition to prevent possible de novo ubiquitination of MSMB during IVC of boar spermatozoa. Our results show that inhibiting neither proteasome nor E1 would result in the accumulation of MSMB in these IVC spermatozoa when compared to the control IVC. These results were obtained consistently by both approaches employed, i.e., flow cytometry as well as Western blotting. Since a protein targeted for degradation via the ubiquitin-proteasome pathway needs to be tagged with a multi-ubiquitin chain of at least four ubiquitin molecules [26], we performed polyubiquitinated protein pulldown using the recombinant UBA domain, hoping to detect polyubiquitinated forms of MSMB. We did not detect MSMB in the fraction of polyubiquitinated proteins. Even with the alternative approach applied, we were still unable to detect ubiquitin in the MSMB immunoprecipitate. We showed that these two strategies were successful in isolating polyubiquitinated proteins and MSMB, respectively. Interestingly, we observed multiple forms of MSMB, i.e., 12, 17, and 22 kDa in both the ejaculated sperm extract and the MSMB immunoprecipitate, in accordance with a previous study [22]. Since the ubiquitin affinity purification studies excluded the possibility that these might be ubiquitinated forms of MSMB, post-translational modifications (PTM) of MSMB other than ubiquitination (e.g., glycosylation) remain to be exposed.

It is very reasonable to conclude that the ubiquitin-proteasome system does not seem to be involved in the degradation of porcine β-microseminoprotein during sperm capacitation, at least not directly as our results show. Image-based flow cytometry and Western blot detection did not prove MSMB accumulation after proteasomal inhibition during IVC. Additionally, we did not find MSMB among ubiquitinated sperm proteins nor observe reduced MSMB degradation during IVC under proteasomal inhibition. The question of what mechanism is responsible for MSMB removal from the sperm surface during capacitation remains to be explored, but it is without a doubt that identification of MSMB PTMs other than ubiquitination would help greatly in such endeavor. Our study presented important information about sperm surface MSMB that opens new avenues for further studies. Altogether, this study and previous studies of UPS-regulated sperm surface proteins indicate that the capacitation-induced processing of sperm surface proteins is more complex than previously thought, employing multiple, non-redundant proteolytic systems.

## 4. Materials and Methods

### 4.1. Semen Collection and Processing

Fresh boar semen was purchased from insemination station Skršín (NATURAL, spol. s.r.o.), and National Swine Research and Resource Center (University of Missouri, Columbia, MO, USA). Approved Animal Care and Use protocols were followed. Concentration and motility of ejaculated spermatozoa were evaluated by conventional spermatological methods under a light microscope. Only ejaculates with ˃80% motile spermatozoa and ˂20% morphological abnormalities were used for the experiment.

Fresh ejaculates were divided into halves, the first half being designated for in vitro capacitation, see below. The second half was washed three times (5 min, 500× *g*) to separate seminal plasma from spermatozoa in warm phosphate-buffered saline (PBS; Sigma-Aldrich, St. Louis, MO, USA), and then spermatozoa were divided into three groups for use in flow cytometric analysis, immunofluorescence staining and protein extraction, as described below. 

### 4.2. Sperm In Vitro Capacitation (IVC) under Proteasomal and E1 Inhibition

To separate them from seminal plasma, fresh, non-extended spermatozoa were washed three times (5 min, 500× *g*) in warm 4-(2-hydroxyethyl)-1-piperazineethanesulfonic acid (HEPES) buffered Tyrode lactate medium supplied with 0.01% (*w*/*v*) polyvinyl alcohol (TL-HEPES-PVA); containing 10 mM Na-lactate; 0.2 mM Na-pyruvate; 2 mM NaHCO_3_; 2 mM CaCl_2_; 0.5 mM MgCl_2_; pH 7.4; 37 °C). After the final wash, spermatozoa were resuspended in TL-HEPES-PVA medium supplied with 2% (*w*/*v*) bovine serum albumin (BSA). Four treatment groups were initiated: (i) without proteasomal or E1 inhibition; (ii) with 100 µM MG132 proteasomal inhibitor (ENZO Life Sciences, Farmingdale, NY, USA) dissolved in dimethylsulphoxide (DMSO; Sigma-Aldrich); (iii) with 50 μM PYR41 E1 ubiquitin-activating enzyme inhibitor (ENZO Life Sciences) dissolved in DMSO; and (iv) 0.1% (*v*/*v*) DMSO vehicle control for both MG132 and PYR41.

All four treatment groups were capacitated for 4 h at 37 °C and 5% (*v*/*v*) CO_2_. After IVC, sperm samples were washed three times in warm PBS and processed for flow cytometric quantification, indirect immunofluorescence, and protein extraction. All applicable international, national, and/or institutional guidelines for the care and use of animals were followed. All studies involving vertebrate animals were completed under the strict guidance of the protocol approved by the Animal Care and Use Committee (ACUC) of the University of Missouri (Animal Welfare Assurance number/ACUC protocol # 9500) and the Guide for the Care and Use of Laboratory Animals (NRC 2011). Boars were maintained under standard husbandry practices at the University of Missouri’s National Swine Research Resource Center (https://nsrrc.missouri.edu/). This article does not contain any studies with human participants performed by any of the authors.

### 4.3. Sample Preparation for Flow Cytometric Analysis

Approximately 1 × 10^6^ washed spermatozoa from each treatment group (ejaculated and IVC spermatozoa, with or without proteasomal/E1 inhibitors including vehicle control) were fixed/permeabilized with 50% ice-cold methanol for 15 min, washed in PBS, and blocked with 5% normal goat serum (NGS; Sigma-Aldrich) in PBS supplemented with 0.1% Triton X-100 (PBST) for 30 min at room temperature. Primary rabbit polyclonal antibody anti-MSMB (1:200 dilution; custom made, see [22]) diluted in PBST with 1% NGS was added to sperm samples and incubated overnight at 4 °C. Negative control with normal rabbit serum was done as previously [25]. The following day, spermatozoa were washed with PBST with 1% NGS and incubated for 40 min at laboratory temperature with secondary antibody goat anti-rabbit conjugated to Cyanine5 (GAR-Cy5; Invitrogen, Carlsbad, CA, USA) diluted 1:150 in PBST with 1% NGS. For acrosome integrity assessment, peanut agglutinin lectin conjugated to Alexa Fluor 488 (PNA-AF488; 1:2500 dilution; Molecular Probes, Eugene, OR, USA) was used, and 4′,6-Diamidino-2-Phenylindole Dilactate (DAPI; 1:1500 dilution; Molecular Probes) was used for DNA staining. Both PNA-AF488 and DAPI were mixed and coincubated with secondary antibody. After incubation, spermatozoa were washed twice with 1% NGS PBST prior to flow cytometry.

### 4.4. Image-Based Flow Cytometry

Fluorescently labeled samples were measured on Amnis FlowSight Imaging Flow Cytometer (AMNIS Luminex Corporation, Austin, TX, USA) as described previously [25,37]. The instrument was equipped with a 20× microscope objective (numerical aperture of 0.9) with an imaging rate of up to 2000 events per second. Sheath fluid was PBS (without Ca^2+^ or Mg^2+^). The flow-core diameter and speed were 10 µm and 66 mm per second, respectively. Raw data were obtained using INSPIRE^®^ software (AMNIS Luminex Corporation, Austin, TX, USA). To produce the highest resolution, the camera setting was at 1.0 µm per pixel of the charge-coupled device. Samples were analyzed simultaneously with four lasers with wavelengths: 405 nm with intensity set to 50 mW, 488 nm with intensity set to 50 mW, 642 nm with intensity set to 20 mW, and 785 nm (side scatter) with intensity set to 5 mW. A total of 10,000 sperm cells were collected per sample. Data analysis of the raw images was accomplished using IDEAS^®^ software (ver. 6.2.64.0, AMNIS Luminex Corporation, Austin, TX, USA). A single-cell population gate was used for histogram display of mean pixel intensities by frequency for the following channels: AF488 (channel 2), DAPI (channel 7), and Cy5 (channel 11). Intensity histograms of individual channels were then used for drawing regions of subpopulations with varying intensity levels and visual confirmation. The intensity of DAPI (channel 7) was used for histogram normalization between different experimental groups.

### 4.5. Indirect Immunofluorescence Imaging

Ejaculated spermatozoa and all treatment sperm groups of IVC spermatozoa were subjected to immunofluorescent imaging using standard procedures [38]. Sperm suspension was adjusted to the concentration of 1 × 10^5^ cells/mL, and sperm smears were prepared. Samples were fixed in cold acetone for 10 min and then washed with PBS. Fixed spermatozoa were incubated with 100 μL of primary rabbit polyclonal antibody anti-MSMB, diluted 1:50 in PBS in a wet chamber at 4 °C overnight. For negative control, sperm samples were incubated only with PBS. After washing with PBS, samples were incubated with 100 μL of secondary anti-rabbit immunoglobulin antibody conjugated with Alexa 488 (Alexa Fluor^TM^488 goat anti-rabbit IgG (H + L), Invitrogen) diluted 1:300 in PBS for 1 h at laboratory temperature. Afterward, samples were incubated with PNA lectin conjugated with Rhodamine (Rhodamine Peanut Agglutin, Vector Laboratories, Burlingame, CA, USA) diluted 1:500 in PBS for 30 min. Samples were mounted with 10 μL of a mounting medium containing DAPI (Vecta-Shield DAPI, Vector Laboratories) and imaged using ZEISS confocal microscope, and ZEN 2.3 software (Zeiss, Jena, Germany).

### 4.6. Sperm Protein Extraction

Prior to protein extraction, all experimental groups (ejaculated and IVC spermatozoa, with or without proteasomal/E1 inhibitors including vehicle control) were washed three times in PBS. Approximately 5 × 10^7^ sperm cells were lysed in 50 µL of twice concentrated reducing loading buffer (0.5 M Tris-HCl pH 6.8 (Bio-Rad, Hercules, CA, USA); glycerol; 2% SDS (sodium dodecyl sulfate); 0.05% bromophenol blue; 5% mercaptoethanol (Sigma-Aldrich)). Samples were kept on ice for 30 min and vortexed every 5 min. Thereafter, sperm samples were boiled for 5 min and centrifuged at 10,000× *g* for 2 min. Sperm protein extracts were subjected to SDS-PAGE (sodium dodecyl sulfate-polyacrylamide gel electrophoresis).

### 4.7. Immunoprecipitation

Polyclonal antibody against MSMB (2 µL; [22]) or rabbit IgG (5 µg; Sigma-Aldrich) for control was added to 100 µL of sperm lysate in IP lysis buffer (ThermoFisher Scientific, Waltham, MA, USA) with a cocktail of protease inhibitors (cOmplete™, Mini; Roche, Basel, Switzerland) and incubated for 1.5 h at 37 °C. Then 50 µL of the agarose beads conjugated with protein A/G (Santa Cruz Biotechnology, Inc., Dallas, TX, USA) were used for protein-antibody complex precipitation. The beads were washed two times with PBS supplemented with 0.1% (*v*/*v*) Tween 20 (PBS-Tween), and bound protein was eluted by boiling the beads for 5 min with reducing SDS loading buffer. The suspension was afterward centrifugated at 10,000× *g* for 3 min, and the supernatant was subjected to SDS-PAGE followed by Western blot immunodetection.

### 4.8. Affinity Isolation of Polyubiquitinated Proteins

Signal-Seeker^TM^ Ubiquitination Detection kit (cat# BK161, Cytoskeleton, Denver, CO, USA) was used to isolate ubiquitinated proteins from spermatozoa according to the manufacturer’s protocol. Briefly, 500 million spermatozoa were lysed with supplied lysis buffer, diluted five times with supplied dilution buffer, and incubated with Ubiquitination Affinity Beads at 4 °C overnight. The following day, the beads were washed with supplied wash buffer and the precipitated polyubiquitinated proteins were eluted by incubating the beads with SDS loading buffer for 5 min. The supernatant was used for Western blot immunodetection. Negative control was performed by incubating the sperm protein lysate with Ubiquitination Control Beads in the same fashion. 

### 4.9. SDS-PAGE and Western Blot

For vertical electrophoresis, a Mini-PROTEAN Tetra system (Bio-Rad) and electrode buffer (25 mM Tris, 192 mM glycine, 0.1% SDS, pH 8.3) were used for sperm protein separation. Sperm samples were run on a 4% stacking and 15% or NuPAGE 4–12% Bis-Tris (Invitrogen) running SDS polyacrylamide gels using Precision Plus Protein™ Dual Color Standards (Bio-Rad) or Novex Sharp Pre-stained Protein standard (ThermoFisher Scientific) as a molecular weight marker. Electrophoresis was run for 20 min at voltage 80 V, and voltage was switched 150 V and run till the leading color band reached the end of gel (about 1.5 h). The proteins were afterward electrotransferred onto a nitrocellulose membrane Hybond^TM^C (Amersham, Little Chalfont, UK) at a constant current of 500 mA for 45 min in Tris-glycine transfer buffer (25 mM Tris, 192 mM glycine, 20% (*v*/*v*) methanol, pH 8.3).

### 4.10. Protein Immunodetection

The nitrocellulose membranes with transferred proteins were blocked for one hour with 5% non-fat milk (Blotting Grade Blocker Non-Fat Dry Milk, Bio-Rad) in PBS-Tween (0.5% Tween 20; Sigma-Aldrich) and incubated in parallel with primary antibodies anti-MSMB (1:500 dilution, polyclonal rabbit antibody) and anti-ubiquitin antibody (FK2, 1:250 dilution, monoclonal mouse antibody recognizing mono- and polyubiquitinated conjugates; ENZO Life Sciences), both in 1% non-fat milk in PBS-Tween, overnight. For protein normalization purposes, the membranes were stripped and incubated with monoclonal antibody anti-alpha-tubulin DM1A (1:5000 dilution; Sigma-Aldrich). The following day, membranes were washed in PBS-Tween and incubated with HRP-conjugated species-specific secondary antibodies such as goat anti-rabbit IgG and goat anti-mouse IgG (1:3000 dilution; Bio-Rad) in 1% non-fat milk in PBS-Tween for 60 min at laboratory temperature. The membranes were washed four times in PBS-Tween and two times in PBS, reacted with a chemiluminescent substrate (Super Signal West Pico Chemiluminescent Substrate; ThermoFisher Scientific), and reactive bands were screened with Azure c600 imaging system (Azure Biosystems). 

### 4.11. Statistical Analysis

All experiments were repeated four times. For all four independent replicates, flow cytometric measurements and immunodetection of transferred proteins were performed. Each data point is presented as mean ± SD. Datasets were tested for normal distribution by the Shapiro-Wilk normality test and processed using the one-way analysis of variance (ANOVA) in a completely randomized design in GraphPad Prism 5 (GraphPad Prism Software, Inc., La Jolla, CA, USA). Tukey post hoc analysis was performed to compare mean values of individual treatment groups with a significance level (alpha) 0.05.

## Supplementary Materials

The following are available online at https://www.mdpi.com/1422-0067/21/11/4151/s1, Figure S1. Localization of porcine MSMB in IVC proteasomally-inhibited (A, A´), and E1-inhibited (B, B´) spermatozoa, and IVC spermatozoa with vehicle control (C, C´) with a specific polyclonal anti-MSMB antibody (green) by indirect immunofluorescent microscopy. Nucleus was counterstained with DAPI (blue) and acrosome with PNA (red). Figure S2. Negative control of MSMB immunoprecipitation (IP MSMB) without antibody and with rabbit immunoglobulins (IP IgG). Ejaculated sperm extract was incubated with agarose-protein A/G beads only; neither MSMB nor polyubiquitinated proteins with FK2 antibody was detected. Asterisks indicate heavy and light chains of immunoglobulins.
