# Supplementary material for: The Ubiquitin-Proteasome System Does Not Regulate the Degradation of Porcine β-Microseminoprotein during Sperm Capacitation
Source: Int J Mol Sci. 2020 Jun 10;21(11):4151. doi: 10.3390/ijms21114151 (PMC7312034; doi:10.3390/ijms21114151)
Supplement: Supplementary file 1 [file ijms-21-04151-s001.pdf]

Supplementary Materials

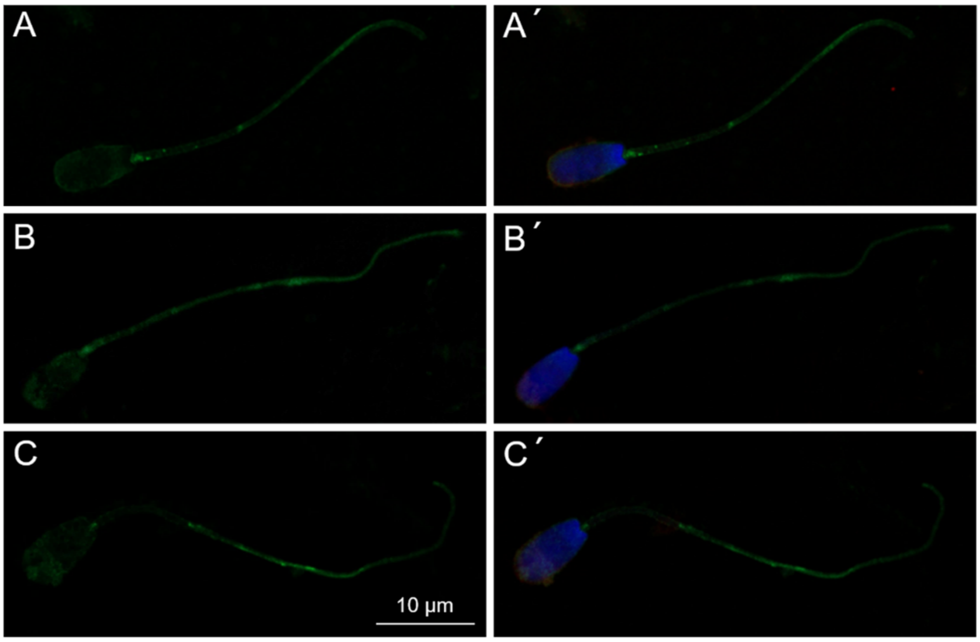

Figure S1. Localization of porcine MSMB in IVC proteasomally-inhibited (A, A´), and E1-inhibited (B, B´) spermatozoa, and IVC spermatozoa with vehicle control (C, C´) with a specific polyclonal anti-MSMB antibody (green) by indirect immunofluorescent microscopy. Nucleus was counterstained with DAPI (blue) and acrosome with PNA (red).

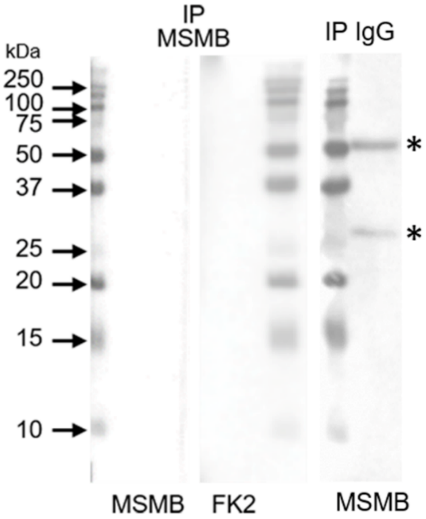

Figure S2. Negative control of MSMB immunoprecipitation (IP MSMB) without antibody and with rabbit immunoglobulins (IP IgG). Ejaculated sperm extract was incubated with agarose-protein A/G beads only; neither MSMB nor polyubiquitinated proteins with FK2 antibody was detected. Asterisks indicate heavy and light chains of immunoglobulins.
